# Evidence-based maintenance care among chiropractors in Norway: a cross-sectional survey in the Nordic maintenance care program

**DOI:** 10.1186/s12998-023-00502-3

**Published:** 2023-08-10

**Authors:** Birgitte Lawaetz Myhrvold, Tora Bjørkmann Vikhaug, Alister DuRose, Anne Marie Gausel, Andreas Eklund, Iben Axén

**Affiliations:** 1 Department of Interdisciplinary Health Sciences, Institute of Health and Society, University of Oslo, Blindern, P.O. Box 1089, 0317 Oslo, Norway; 2 Et Liv I Bevegelse, The Norwegian Chiropractic Research Foundation, Oslo, Norway; 3 AECC University College, Parkwood Campus, Parkwood Road, Bournemouth, UK; 4 Stavanger University Hospital, Stavanger, Norway; 5 Department for Caring and Ethics, Faculty of Health Sciences, University of Stavanger, Stavanger, Norway; 6 Unit of Intervention and Implementation Research for Worker Health, Institute of Environmental Medicine, Karolinska Institutet, Nobels Väg 13, 171 77 Stockholm, Sweden

**Keywords:** Evidence-based, Maintenance, Attitudes, Skills, Use, Facilitators, Barriers

## Abstract

**Background:**

Chiropractors use a treatment strategy called maintenance care with the intent of secondary and tertiary prevention. The Nordic Maintenance Care Program provides evidence of when and for whom maintenance care should be offered. Dissemination has occurred through articles, workshops, social media, conference in Europe and internationally. However, little is known about chiropractors’ awareness and use of this evidence. This study explores chiropractors’ attitudes, skills, and use of evidence on maintenance care, as well as study their association with general evidence-based practice and demographic characteristics. Moreover, barriers and facilitators of evidence access are also examined.

**Methods:**

Questions pertaining our research aim were included in the anonymous and digital Evidence-Based practice Attitude and utilization SurvEy, which was distributed to all members (n = 770) of the Norwegian chiropractic association in the fall of 2021.

**Results:**

The response rate was 41% (n = 312). Regarding attitudes towards evidence-based maintenance care, 26% agreed they needed tools to recommend this care to patients. Approximately half (57%) believed they had *skills* to identify suitable patients, and 45% had *used* published information in the past month. Strong alignment was observed between Norwegian chiropractors’ attitudes, skills, and utilization of evidence-based maintenance care and general evidence-based practice. Maintenance care *skills* were significantly associated with age (those between 40 and 59 years being less likely to report having high skills), clinical setting (those working with conventional health care providers being less likely to report having high skills) and country of education (those educated in the US and Australia being more likely to report having high skills). Moreover, maintenance care *use* was significantly associated with country of education (those educated in Australia were less likely to have used published information regarding patient selection for maintenance care). Access to resources was a barrier, whereas knowledge of patient suitability facilitated evidence-based maintenance care.

**Conclusions:**

Norwegian chiropractors had neutral attitudes towards maintenance care, but generally reported moderate skills. Most had not used evidence about maintenance care in the previous month. Access to useful resources about the evidence regarding maintenance care was a barrier, and knowledge of who responds to maintenance care was a facilitator.

**Supplementary Information:**

The online version contains supplementary material available at 10.1186/s12998-023-00502-3.

## Background

Chiropractors traditionally use a secondary and tertiary preventive strategy known as Maintenance Care (MC) to manage recurrent and persistent musculoskeletal pain [1]. Maintenance care may be defined as treatment that continues after optimum benefit is reached, regularly regardless of symptoms [2]. MC is used to reduce the impact of recurrent pain and to minimize the consequences of episodic or persistent (chronic) pain [1]. From a public health standpoint, it makes sense to use preventive efforts when treating such conditions [3], as their etiology is largely unknown, and no cure exists.

During the past 10 years, a research program in the Nordic countries has investigated the concept of MC: its prevalence, indications, and effect [1]. Specifically, we know that patients with recurrent and persistent low back pain who respond well to an initial treatment course AND have a ‘dysfunctional’ psychological profile (high pain severity which interferes with everyday life, high affective distress, low perception of life control and low activity levels), respond well to MC [4, 5]. In a recent randomized controlled trial, these patients experienced fewer days with bothersome low back pain at the same number of treatments as patients who were told to come for care when needed [5]. In contrast, patients who are classified as ‘adaptive copers’ (low pain severity, low interference with everyday life, low life distress, a high activity level, and a high perception of life control) fare worse on MC and should not be treated with this strategy [4].

Work in the Nordic Maintenance Care Program is ongoing to develop stratified care pathways and implement the knowledge acquired from the past decade of research. The psychological profile tool used to identify patients with likely positive and negative outcomes from MC (the Multidimensional Pain Inventory) is a comprehensive questionnaire needing statistical software to be scored according to a complex clustering procedure and not very clinic-friendly [6]. At the time of this survey, a tool to identify patients appropriate for MC was being developed and tested and has recently been published for clinical use [7]. However, in clinical practice, the profiles may be ‘recognizable’ as distinct patient types, providing the chiropractor is aware of what to look for, i.e., is familiar with the research.

In Scandinavia and Europe, the results from the Nordic Maintenance Care program have been communicated in short videos that have been published in specific social media forums, in podcasts and interviews, as well as in workshops and lectures online and in person (for an overview, please see Additional File 1). In Norway particularly, the findings were presented at the yearly chiropractic conference in October 2018, as well as in newsletters from the Norwegian research foundation with every new publication of the program thereafter. Moreover, the results have been presented at the World Federation of Chiropractic and European Chiropractors’ Union conferences, where two research prizes have been awarded. Thus, the research findings have been given much attention and debated on internet forums and social media.

There is some evidence that chiropractors in the Nordic countries have positive attitudes toward Evidence-Based Practice (EBP), i.e. the integration of the best available research evidence into clinical practice to improve both health outcomes and quality of care for individual patients [8, 9]. To target future implementation strategies related to MC research evidence for chiropractors, we wanted to explore chiropractors’ attitudes, their skills, and if they use available evidence on MC. Moreover, if barriers and facilitators of access to such evidence exist.

This knowledge is necessary if optimal patient management through the utilization of EBP is the goal. Thus, the results may influence future research communication and implementation concerning MC evidence.

The aim of this study was to explore chiropractors' attitudes, skills, and use of available evidence on maintenance care and to study if these are associated with attitudes, skills, and use of evidence in general and with demographic factors. Moreover, if barriers and facilitators of access to such evidence exist.

## Methods

The objectives of the study were to explore the following questions:

1. What are chiropractors' attitudes towards the usefulness of resources/tools for prescribing MC in clinical practice?
2. Do chiropractors think they have the skills to identify patients likely to respond positively or negatively to MC?
3. Do chiropractors often use published information to identify patients suitable for MC?
4. Do chiropractors consider that barriers exist regarding access to useful resources to evaluate if MC is appropriate?
5. Do chiropractors believe it is a facilitator to have knowledge of which patients are likely to respond positively or negatively to MC?
6. What are the associations between attitudes, skills, and use of EBP in general and attitudes, skills, and use of evidence relating to MC?
7. . What are the associations between attitudes, skills, and use of evidence relating to MC and the chiropractor’s demographic factors?

### Design and ethics

The study was based on data from an online, cross-sectional survey conducted in Norway. No identifying data were collected, and results were reported as aggregated data, thus maintaining participant anonymity. Study participation was voluntary, and no ethical approval was needed according to Norwegian law [10]. The study was carried out in accordance with ethical guidelines; informed consent was obtained from all participants in the digital questionnaire.

### Setting and participants

All chiropractors (n = 770) registered as members of the Norwegian Chiropractors’ Association in 2021 were eligible and invited to participate. In Norway, this is approximately 90% of all licenced chiropractors [11].

### Data collection procedures

In the fall of 2021, a questionnaire survey was conducted using an online system called 'Nettskjema' developed at the University of Oslo, Norway. The survey link was distributed via email to 770 practicing chiropractors who were members of the Norwegian Chiropractors' Association. Various methods were used to encourage participation, including social media posts, emails, and personal reminders. Verbal advertisements and encouragement were also provided during the National Norwegian chiropractic Conference. Data collection took place between October and November 2021, and participants were provided with information on the study's purpose, the meaning of their consent, data storage, reporting of results, and ethical considerations. Multiple reminders and messages were sent through email and social media groups to encourage completion of the survey.

### Description of the questionnaire and variables

The questions for this study (described below) were added to a more extensive questionnaire called The Evidence-Based practice Attitude and utilization SurvEy (EBASE), which has shown good internal consistency, construct and content validity and demonstrated acceptable test–retest reliability [12, 13]. The results of this study have been presented elsewhere [14]. EBASE is an instrument that evaluates attitudes, perceived skills, and the use of evidence-based practice among healthcare providers. EBASE is divided into seven parts: attitudes (part A), skill level (part B), education and training (part C), use (part D), barriers to EBP (part E), and enablers of EBP (part F). The final section, part G, contains participant demographics. For this study, parts A, B, D, and G of EBASE were used, exploring attitudes skills and the use of EBP in general.

**Table:** 

Part A: attitudes towards EBP (10 items, rated using a 5-point scale, ranging from ‘Strongly disagree’ to ‘Strongly agree’),
Part B: skills of EBP (13 items, rated using a 5-point scale, ranging from ‘Low’ to ‘High’)
Part D: use of EBP (9 items, rated based on the number of articles read/reviewed, number of times performing certain EBP-related activities, and information sources used to inform clinical decision-making)
Part G: demographics (14 multiple-choice items and one open-text item)

For parts A, B, and D of EBASE, scores were generated and dichotomized for objective six as follows:

A. EBP attitude; scores ranging from 8 (predominantly ‘strongly disagree’) to 40 (predominantly ‘strongly agree’); the subgroups were labelled ‘Disagree’ (sub-score < 32) and ‘Agree’ (sub-score >  = 32),
B. EBP skills; scores ranging from 13 (primarily ‘low-level skill’) to 65 (primarily ‘high-level skill’); the subgroups were labelled ‘Poor-average’ (sub-score <  = 39) and ‘Average-good’ (sub-core > 39), and.
C. : EBP use; scores ranging from 0 (mainly ‘infrequent use’) to 24 (mainly ‘frequent use’); the subgroups were labelled ‘Never-rarely’ (sub-score <  = 8) and ‘Sometimes’ (sub-core > 8).

Part G of EBASE were used to describe the study participants, and to investigate associations between MC attitudes, MC skills and MC use and demographic factors for objective 7. The selection of candidate factors to investigate associations was based on the literature of identified demographic factors [15, 16]. The following factors were considered: gender, age, number of years in practice, clinical setting, and country of education.

For this study, we included five specific questions and answer options specifically pertaining to MC:

**Table:** 

1: MC attitudes: Use of instruments (such as West Haven-Yale Multidimensional Pain Inventory or STarT back) are useful tools in my profession for appropriate prescription of maintenance care Answer options: Strongly disagree, Disagree, Neutral, Agree, Strongly agree
2: MC skills: I have sufficient skills to identify the patients which responds positively or negatively to maintenance care Answer options: Low skills, Low-medium skills, Medium skills, Medium–high skills, High skills
3: MC use: I have used published information to find the appropriate patient group for which maintenance care is recommended Answer options: How often during the past month: 0 times, 1–5, 6–10, 11–15, 16 + times
4: MC barriers: Access to useful resources (e.g., questionnaires, relevant articles) for subgrouping of patients to evaluate if maintenance care is appropriate is: Answer options: Not a barrier, A minor barrier, A moderate barrier, A major barrier
5: MC facilitators: Having the knowledge of which patient groups respond positively or negatively to maintenance care is: Answer options: Not useful, Slightly useful, Moderately useful, Very useful

For objective 7, the responses to the questions regarding MC attitudes, MC skills and MC use items were dichotomized.

1. MC attitudes were dichotomized into ‘Disagree’ (including the response options ‘Strongly disagree’, ‘Disagree’ and ‘Neutral’), and ‘Agree’ (including the responses ‘Agree’ and ‘Strongly agree’).
2. MC skills were dichotomized into ‘Low skills’ (including the response options ‘Low Skills’, ‘Low-medium’ and ‘Medium’) and ‘High skills’ (including the responses ‘Medium–high’ and ‘High Skills’).
3. MC use were dichotomized into ‘no use’ (including response options ‘0 times’) and ‘use’ (including the ‘1–5’, ‘6–10’, ‘11–15’, ‘16 + times’ responses).

### Statistical methods

Survey responses were exported directly into and analyzed with STATA/SE 16 (STATA Corp, College Stations, TX). There were no missing data, as all items were made compulsory.

Categorical data were described using frequency distributions and percentages. Cross-tables were used to analyze the association between EBP attitudes, EBP skills, and EBP use and MC attitudes, MC skills, and MC use.

The associations between MC attitudes, MC skills, and MC use as outcomes and the selected demographics as independent variables were investigated by logistic regression analysis. The result is presented as odds ratios (OR) with a 95% confidence interval.

## Results

### Description of the sample

The response rate for the survey was 41% (n = 312) of the target sample. Of the participating chiropractors, 174 (56%) were male (Table 1). Most participating chiropractors (70%) were in the age range 30–50 years, and approximately half (55%) had been in practice for more than 11 years. Most of the chiropractors graduated from a chiropractic college in Great Britain (62%), and many chiropractors (66.5%) held a bachelor’s or higher-level graduate degree. Most (68%) worked in a clinical setting with conventional healthcare providers, and only 8% were in solo practice. For detailed demographic data, see Table 1.

**Table 1:** Demographic characteristics of the sample (n = 312)

Characteristics	Frequency, n (%)
*Age*	
20–29 years	41 (13)
30–39 years	129 (41)
40–49 years	90 (29)
50–59 years	36 (12)
60 + years	16 (5)
*Sex*	
Male	174 (56)
Female	134 (43)
Do not wish to state	4 (1)
*Highest qualification*	
University or College Certificate/Diploma	99 (32)
Bachelor’s or Master’s degree (2 years)	203 (65)
PhD/Doctorate	5 (1.5)
Other	5 (1.5)
*Years practiced in the field of chiropractic*	
0 years	11 (4.5)
1–5 years	46 (15)
6–10 years	82 (26)
11–15 years	71 (22.5)
16 + years	102 (33)
*Clinical setting in which chiropractic is predominantly practiced*	
Solo practice	26 (8)
With a group of chiropractors	63 (20)
With a group of conventional health providers	140 (45)
With a group of CM health providers	10 (3)
With CM & conventional health providers	73 (23)
*Country of education*	
Great Britain	193 (62)
Denmark	52 (17)
USA	38 (12)
Australia	28 (9)

CM = Complementary Medicine

The results of the EBP attitudes, EBP skills and EBP use have been presented in a separate publication [14].

The answers to the questions pertaining to MC are presented in Table 2. Few chiropractors (26%) agreed that the use of tools to recommend MC to patients in clinical practice is useful. Approximately half (57%) believed they had sufficient skills to identify the patients who are likely to respond positively or negatively to MC. However, only 45% of the participating chiropractors reported having used published information to find the appropriate patient group for which MC is recommended, in the past month. Regarding useful resources to evaluate if MC is appropriate, the majority (80%) believed that limited access to useful resources is a barrier. Almost all (97%) reported that knowledge of which patient groups respond to MC is useful.

**Table 2:** Knowledge and use of Evidence-Based Care pertaining to maintenance care among Norwegian chiropractors (n = 312)

Domain	Frequency, n (%)
*MC attitude*	
Use of instruments (such as West Haven-Yale Multidimensional Pain Inventory or STarT back) are useful tools in my profession for appropriately prescription on maintenance care	
Strongly disagree	21 (7)
Disagree	61 (20)
Neutral	**150 (48)**
Agree	65 (21)
Strongly agree	15 (5)
*MC skill*	
I have sufficient skills to identify the patients which responds well and poorly to maintenance care	
Low skills	15 (5)
Low-medium skills	25 (8)
Medium skills	97 (31)
Medium–high skills	**136 (44)**
High skills	39 (13)
*MC use*	
I have used published information to find the appropriate patient group for which maintenance care is recommended	
0 times	**172 (55)**
1–5 times	87 (28)
6–10 times	23 (7)
11–15 times	12 (4)
16 + times	18 (6)
*MC barriers*	
Access to useful resources (e.g., questionnaires, relevant articles) to subgroup patients to evaluate if maintenance care is appropriate is…	
Not a barrier	61 (20)
A minor barrier	**128 (41)**
A moderate barrier	97 (31)
A major barrier	26 (8)
*MC facilitators*	
Having knowledge of which patient groups responds positively or negatively to maintenance care is…	
Not useful	13 (4)
Slightly useful	33 (11)
Moderately useful	92 (30)
Very useful	**174 (56)**

*Main responses in bold*

Only small dissimilarities existed between chiropractors’ EBP attitudes, EBP skills, and EBP use and their MC attitudes, MC skills, and MC use.

Regardless of EBP attitudes, chiropractors’ MC attitudes towards the use of instruments were largely ‘neutral’ (between 45 and 50%), see Table 3. However, 63% of the chiropractors who agree that EBP is necessary (EBP attitude), reported that it is ‘very useful’ to have the knowledge of which patient group responds to MC (MC facilitator), but only 40% among those who disagree that EBP is necessary thought so. Regarding MC skills, MC use, and MC barriers, no major differences were observed in the two EBP attitudes subgroups.

**Table 3:** Cross tabulations of MC Attitudes, MC Skills, MC Use, MC Barriers, and MC Facilitators and the EBP Attitudes, EBP Skills and EBP Use

	EBP Attitudes	
	Disagree (sub-score < 32)	Agree (sub-score > = 32)
	N = 104	N = 208
*MC attitudes, n (%)*		
Use of instruments (such as West Haven-Yale Multidimensional Pain Inventory or STarT back) are useful tools in my profession for appropriately prescription on maintenance care		
Strongly disagree	5 (5)	16 (8)
Disagree	35 (34)	26 (13)
Neutral	**47 (45)**	**103 (50)**
Agree	16 (15)	49 (24)
Strongly agree	1 (1)	14 (7)
*MC skills, n (%)*		
I have sufficient skills to identify the patients which responds well and poorly to maintenance care		
Low skills	5 (5)	10 (5)
Low-medium skills	7 (7)	18 (9)
Medium skills	32 (31)	65 (31)
Medium–high skills	**47 (45)**	**89 (43)**
High skills	13 (13)	26 (13)
*MC use, n (%)*		
I have used published information to find the appropriate patient group for which maintenance care is recommended		
0 times	**58 (56)**	**114 (55)**
1–5 times	33 (32)	54 (26)
6–10 times	5 (5)	18 (9)
11–15 times	4 (4)	8 (4)
16 + times	4 (4)	14 (7)
*MC barriers, n (%)*		
Access to useful resources (e.g., questionnaires, relevant articles) to implement subgrouping of patients before one can evaluate if maintenance care is appropriate is:		
Not a barrier	18 (17)	43 (21)
A minor barrier	**44 (42)**	**84 (40)**
A moderate barrier	32 (31)	65 (31)
A major barrier	10 (10)	16 (8)
*MC facilitators, n (%)*		
Having the knowledge of which patient groups responds positively or negatively to maintenance care is:		
Not useful	5 (5)	8 (4)
Slightly useful	13 (13)	20 (10)
Moderately useful	**44 (42)**	48 (23)
Very useful	42 (40)	**132 (63)**

	EBP Skills	
	Poor-average (sub-score < = 39)	Average-good (sub-score > 39)
	N = 143	N = 169
*MC attitudes, n (%)*		
Use of instruments (such as West Haven-Yale Multidimensional Pain Inventory or STarT back) are useful tools in my profession for appropriately prescription on maintenance care		
Strongly disagree	8 (6)	13 (8)
Disagree	38 (27)	23 (14)
Neutral	**66 (46)**	**84 (50)**
Agree	26 (18)	39 (23)
Strongly agree	5 (4)	10 (6)
*MC skills, n (%)*		
I have sufficient skills to identify the patients which responds best and worst to maintenance care		
Low skills	7 (5)	8 (5)
Low-medium skills	14 (10)	11 (7)
Medium skills	39 (27)	58 (34)
Medium–high skills	**75 (52)**	**61 (36)**
High skills	8 (6)	31 (18)
*MC use, n (%)*		
I have used published information to find the appropriate patient group for which maintenance care is recommended		
0 times	**83 (58)**	**89 (53)**
1–5 times	47 (33)	40 (24)
6–10 times	5 (2)	18 (11)
11–15 times	6 (4)	10 (6)
16 + times		12 (7)
*MC barriers, n (%)*		
Access to useful resources (e.g., questionnaires, relevant articles) to implement subgrouping of patients before one can evaluate if maintenance care is appropriate is:		
Not a barrier	24 (17)	37 (22)
A minor barrier	50 (35)	**78 (46)**
A moderate barrier	**54 (38)**	43 (25)
A major barrier	15 (10)	11 (7)
*MC facilitators, n (%)*		
Having the knowledge of which patient groups responds positively or negatively to maintenance care is:		
Not useful	4 (3)	9 (5)
Slightly useful	11 (8)	22 (13)
Moderately useful	46 (32)	46 (27)
Very useful	**82 (57)**	**92 (54)**

	EBP Use	
	Never-rarely use (sub-score < = 8)	Sometimes use (sub-score > 8)
	N = 172	N = 140
*MC attitudes, n (%)*		
Use of instruments (such as West Haven-Yale Multidimensional Pain Inventory or STarT back) are useful tools in my profession for appropriately prescription on maintenance care		
Strongly disagree	9 (5)	12 (9)
Disagree	39 (23)	22 (16)
Neutral	**78 (45)**	**72 (51)**
Agree	39 (23)	26 (19)
Strongly agree	7 (4)	8 (6)
*MC skills, n (%)*		
I have sufficient skills to identify the patients which responds best and worst to maintenance care		
Low skills	5 (3)	10 (7)
Low-medium skills	15 (9)	10 (7)
Medium skills	52 (30)	45 (32)
Medium–high skills	**84 (49)**	**52 (37)**
High skills	16 (9)	23 (16)
*MC use, n (%)*		
I have used published information to find the appropriate patient group for which maintenance care is recommended		
0 times	**106 (62)**	**66 (47)**
1–5 times	57 (33)	30 (21)
6–10 times	6 (3)	17 (12)
11–15 times	1 (1)	11 (8)
16 + times	2 (1)	16 (11)
*MC barriers, n (%)*		
Access to useful resources (e.g., questionnaires, relevant articles) to implement subgrouping of patients before one can evaluate if maintenance care is appropriate is:		
Not a barrier	30 (17)	31 (22)
A minor barrier	**75 (44)**	**53 (38)**
A moderate barrier	53 (31)	44 (31)
A major barrier	14 (8)	12 (9)
*MC facilitators, n (%)*		
Having the knowledge of which patient groups responds positively or negatively to maintenance care is:		
Not useful	8 (5)	5 (4)
Slightly useful	15 (9)	18 (13)
Moderately useful	51 (30)	41 (29)
Very useful	**98 (57)**	**76 (54)**

In the EBP skills subgroups, among chiropractors who rated their skills as ‘average-good’, access to instruments to subgroup patients suitable for MC were mainly found to be a minor MC barrier, whereas those rating their EBP skills as ‘poor-average’, found it to be a moderate barrier, see Table 3. Regarding MC attitudes, MC skills, MC use and MC facilitators, no major differences were observed in the EBP skills subgroups.

In the EBP use subgroups, more chiropractors reporting to use EBP ‘sometimes’ compared to those reporting ‘never-rarely’ answered that they had used scientific literature during the past month (MC use), see Table 3. No major differences were observed in the EPB use subgroups concerning MC attitudes, MC skills, MC barriers and MC facilitators.

Logistic regression analysis showed that MC attitudes were not significantly associated with any of the demographic factors (Table 4).

**Table 4:** Associations between MC attitude, MC skills, and MC use and demographic variables

	MC attitudes		MC skills		MC use	
	OR	95% CI	OR	95% CI	OR	95% CI
*Gender*						
Ref women	1.19	(0.70 to 2.04)	0.90	(0.57 to 1.47)	1.62	(1.00 to 2.62)
*Age*						
20–39	Ref					
40–59	1.48	(0.50 to 4.39)	0.37*	(0.14 to 0.96)	0.88	(0.35 to 2.21)
60 +	4.24	(0.82 to 1.72)	0.53	(0.11 to 2.69)	1.09	(0.24 to 4.93)
*Years in practice*						
0–5	Ref					
6–10	0.55	(0.25 to 1.21)	1.02	(0.50 to 2.11)	0.94	(0.46 to 1.94)
11–15	0.42	(0.15 to 1.13)	1.68	(0.70 to 4.03)	0.74	(0.31 to 1.74)
16 +	0.47	(0.13 to 1.75)	2.98	(0.92 to 9.61)	0.48	(0.15 to 1.52)
*Clinical setting*						
Solo	Ref					
w/chiropractors	0.96	(0.32 to 3.04)	0.70	(0.25 to 2.01)	1.22	(0.45 to 3.30)
w/conventional health providers	1.22	(0.44 to 3.39)	0.36*	(0.14 to 0.93)	0.86	(0.35 to 2.12)
w/CM	1.47	(0.27 to 7.69)	0.47	(0.10 to 2.27)	0.87	(0.18 to 4.22)
w/CM and conventional health providers	1.00	(0.34 to 2.98)	0.45	(0.17 to 1.24)	1.06	(0.41 to 2.78)
*Country of education*						
England	Ref					
Denmark	0.82	(0.37 to 1.83)	0.78	(0.39 to 1.56)@	0.69	(0.34 to 1.37)
USA	0.58	(0.22 to 1.55)	2.64*	(1.04 to 6.66)	0.55	(0.23 to 1.30)
Australia	0.63	(0.21 to 1.84)	3.08*	(1.17 to 8.12)	0.34*	(0.13 to 0.85)
*Constant*	0.39	(0.10 to 1.54)	2.61	(0.73 to 9.37)	0.70	(0.20 to 2.40)

CM = Complementary Medicine

The 95% CI not crossing 1 are marked with a * (implying a significant difference in outcome)

MC skills were significantly associated with age, clinical setting, and country of education. Thus, chiropractors in the age range 40–59 who were working with conventional health providers were less likely, in contrast to those educated in the USA and Australia that were more likely, to report that they have ‘medium’ to ‘medium–high’ skills to identify patients that respond positively or negatively to MC (Table 4).

MC use was significantly associated with the country of education; chiropractors educated in Australia were less likely to report that they have used published information to find the appropriate patient group for which MC is recommended (Table 4).

## Discussion

To the best of our knowledge, this is the first study that explicitly examines chiropractors’ knowledge and use of evidence of a specific topic: MC.

We found that most chiropractors had neutral attitudes towards MC. They felt confident in their skills and use towards identifying patients suitable for MC. However, only a small number of chiropractors reported reading research on MC in the previous month. Further, access to useful resources was found to be a barrier, and knowledge of the research findings was a facilitator for identifying suitable patients.

Chiropractors’ perceptions towards EBP were generally good (2/3 reporting to agree that EBP is important [14] but were mainly ‘neutral’ in their attitudes towards evidence in MC. One interpretation is that the neutral attitude towards EBP and evidence in the specific field of MC is a sign of chiropractors not knowing how to respond or not caring about the response. We might speculate that it is the ‘easy’ option that does not require much thought or interest. Still, it may well reflect the issue's complexity, where chiropractors are forced to balance their clinical experience and awareness of the available evidence. Another perspective is that the limited awareness of research findings among chiropractors and their low access to such information may indicate a failure of current dissemination efforts. Despite efforts to disseminate research in the field, only a small number of chiropractors appeared to be aware of the research conducted and few access its findings.

Norwegian chiropractors were confident in their own skills relating to identifying patients suitable for MC. As the tool designed to reliably do this was just recently published, this answer may simply reflect their clinical experience, rather than an evidence-based approach.

The use of evidence pertaining to MC was low. This finding is not surprising, as the same was reported for the general use of EBP [14]. These findings highlight the importance to explore and develop strategies to improve chiropractors’ skills and use regarding published evidence, specifically in identifying patients suitable for and likely to respond to MC. There is a clear need to develop effective approaches aimed at improving these aspects within chiropractic practice.

The attitudes, skills, and use of evidence regarding MC were generally not associated with demographic variables, although the clinical setting and the country of education were significantly associated with MC skills. Chiropractors working in an environment with conventional healthcare providers rated their MC skills as low. Maybe these practitioners are aware of the EBP complexity and feasibility of practice using an evidenced-based paradigm, i.e., what it entails to practice according to evidence, as they are constantly discussing with other healthcare providers and therefore answer truthfully that they need to be more skilled in this matter. Chiropractors practising in a solo practice, educated in the US and Australia, were confident regarding their MC skills. Working alone may not present opportunities to discuss EBP with peers and may result in being ‘over-confident’. Certain educational institutions in the US have a stronger tradition of working with MC [17]. Still, most of the evidence in the field has only been produced in recent years, and the traditional approach is not likely among these educational institutions from an evidence-based perspective. Previous EBASE surveys have identified a high degree of traditional knowledge in the US [18] and Canada [19], and MC may be used within this concept. Those educated in Australia had high confidence in MC skills but amounted only to 9% of the sample. Care should therefore be taken when generalizing these findings.

The many different dissemination activities that have been undertaken to translate the findings of the Nordic Maintenance Care Programme have yet to be successful among Norwegian chiropractors. Only a few efforts were directly targeted at this population, but Norwegian chiropractors are part of European networks where several efforts have been employed. We must work on different fronts: we need to evoke interest (as many rated their attitudes as neutral), providing information through channels other than those tried already (as access to information was considered a barrier). Educational programs at schools should be targeted for this knowledge to be translated to students.

The response rate was only 41%, but still better than that of a recent equivalent Swedish study in the EBP field [8]. It is generally challenging to get a good response rate on surveys, even though we employed several reminder strategies. The low response rate may limit the generalizability of our findings. Further, chiropractors who are positive towards EBP may be more likely to answer a survey such as this. If so, the dissemination of the MC research may have been even less than reported here.

This EBASE study is a direct replica of a recently conducted Swedish study among manual therapists [8], and similar studies have been undertaken among chiropractors in Australia [20], the US [21], and Canada [22]. However, these studies did not incorporate questions concerning attitudes and the use of evidence relating to MC; thus, the evidence base of MC has yet to be tested. Therefore, the participating chiropractors may have interpreted these questions differently than intended, rendering our results uncertain.

## Conclusions

Norwegian chiropractors generally had neutral attitudes towards the tools available to utilize the evidence of MC; they reported moderate skills, and most had not used evidence about MC the previous month. Access to useful resources about evidence of MC was a barrier for most chiropractors, although most thought that knowledge of responses to MC was a facilitator.

### Supplementary Information

**Additional file 1. **Communication of the Nordic Maintenance Care program.

## Data Availability

Data may be shared with other researchers upon reasonable request. The Norwegian questionnaire is available from the authors upon request.

## Abbreviations

MC: Maintenance care
EBP: Evidence-based practice
EBASE: Evidence-Based practice Attitude and utilization SurvEy
CM: Complementary medicine
