# Supplementary material for: Evidence-based maintenance care among chiropractors in Norway: a cross-sectional survey in the Nordic maintenance care program
Source: Chiropr Man Therap. 2023 Aug 10;31:26. doi: 10.1186/s12998-023-00502-3 (PMC10416359; doi:10.1186/s12998-023-00502-3)
Supplement: Supplementary file 1 — Additional file 1. Communication of the Nordic Maintenance Care program. [file 12998_2023_502_MOESM1_ESM.docx]

**Additional file 1: Communication of the Nordic Maintenance Care program**

The following information has been published on the main authors' (Andreas Eklund, AE) social media accounts. On Facebook the information has also been published in 6 different profession-specific groups/forums the main author follows. One of these forums is “Kiropraktikk i Norge” with 997 members (230713) a profession specific group for Chiropractors in Norway.

<https://www.facebook.com/groups/kiropraktikk>

In this forum, AE has posted 9 times, disseminating the 5 main papers in different ways, ranging from one long video (2 hours, reviewing the whole research program), to short 1-2 minute videos relating to specific publications and text.

<https://www.facebook.com/groups/366610736840166/user/676576590>

Following is a list of dates and references for the overall dissemination of the 5 publications on Facebook, Twitter, and LinkedIn; this list does not represent the total number of posts but rather dates of when the different channels were used. The different posts on social media have generated a considerable amount of discussion on the world wide web, and the main author has spent approximately a total of 100 hours posting content and responding to comments between 2018-2023.

230428 Facebook, repost RSS education review of MAINTAIN instrument (1).

220322 Facebook, Repost CMT MAINTAIN instrument (1)

220318 Facebook, MAINTAIN instrument publication (1).

220318 Twitter MAINTAIN instrument publication (1).

210907 Facebook, MAINTAIN publication (preprint) (1).

210907 Twitter MAINTAIN publication (preprint) (1).

210825 Twitter patient experiences publication (2).

210824 LinkedIn, patient experiences publication + video (2).

210824 Facebook, patient experiences publication + video (2).

200422 LinkedIn mechanism publication + video (3).

200422 Twitter mechanism publication + video (3).

200422 Facebook mechanism publication + video (3)

200421 Facebook, WFC repost, mechanism publication + video (3).

191023 Facebook psych factors publication (4).

191011 LinkedIn psych factors publication + video (4).

191011 Facebook psych factors publication + video (4).

191011 Twitter psych factors publication (4).

190705 Facebook mechanism conference presentation (3).

190705 Twitter mechanism conference presentation (3).

181025 Facebook, RSS retweet, review RCT main findings publication (5)

180929 Facebook RCT main findings publication (5)

180919 Facebook RCT main findings publication (5)

180914 Facebook RCT main findings publication + video (5)

180913 Facebook RCT main findings publication (5)

180912 Facebook RCT main findings publication (5)

180912 Twitter RCT main findings publication (5)

Data has also been disseminated at international conferences which Norwegian chiropractors frequently attend.

Eklund A, Palmgren P, Jakobsson U, Axén I. *Maintain, a short clinical instrument for the identification of dysfunctional patients suitable for Maintenance Care.* WFC convention, online, 2021.

Eklund A, Hagberg J, Jensen I, Leboeuf-Yde C, Kongsted A, Lövgren P, Jonsson M, Petersen-Klingberg J, Calvert C, Axén I. *The Nordic Maintenance Care program: How does Maintenance Care work?* Platform presentation, WFC convention, Berlin, Germany 2019. (Winner of the Scott Haldeman Award for Outstanding Research)

Eklund A, Jensen I, Leboeuf-Yde C, Kongsted A, Axén I. *Is effectiveness of Chiropractic Maintenance Care moderated by psychological profile? A secondary analysis of a pragmatic randomized controlled trial*. Platform presentation ECU convention, Budapest Hungary 2018.

Eklund A, Jensen I, Lohela-Karlsson M, Hagberg J, Bodin L, Lebouf-Yde C, Kongsted A, Axén I. *Prevention of low back pain: effect of chiropractic maintenance care as compared to symptomatic treatment – a pragmatic randomized clinical trial.* Researcher speed presentation, Canadian Chiropractic Association’s National Convention and Tradeshow, Calgary Canada 2018.

Eklund A, Jensen I, Lohela-Karlsson M, Hagberg J, Bodin L, Lebouf-Yde C, Kongsted A, Axén I. *Prevention of low back pain: effect of chiropractic maintenance care as compared to symptomatic treatment – a pragmatic randomized clinical trial.* Platform presentation by Iben Axen, ECU convention, Limasol Cyprus 2017, winner of EAC Jean Robert research award (1st prize).

Eklund A, Jensen I, Lohela-Karlsson M, Hagberg J, Bodin L, Lebouf-Yde C, Kongsted A, Axén I. *Prevention of low back pain: effect and cost-effectiveness of preventive treatment (Chiropractic Maintenance Care) as compared to symptomatic treatment – a pragmatic randomized clinical trial.* Platform presentation, WFC convention, Washington USA 2017.

References:

1.      Eklund A, Palmgren PJ, Jakobsson U, Axen I. Development and evaluation of the MAINTAIN instrument, selecting patients suitable for secondary or tertiary preventive manual care: the Nordic maintenance care program. Chiropr Man Therap. 2022;30(1):15.

2.      Hjertstrand J, Palmgren PJ, Axen I, Eklund A. The nordic maintenance care program: patient experience of maintenance care-a qualitative study. Chiropr Man Therap. 2021;29(1):28.

3.      Eklund A, Hagberg J, Jensen I, Leboeuf-Yde C, Kongsted A, Lovgren P, et al. The Nordic maintenance care program: maintenance care reduces the number of days with pain in acute episodes and increases the length of pain free periods for dysfunctional patients with recurrent and persistent low back pain-a secondary analysis of a pragmatic randomized controlled trial. Chiropractic & Manual Therapies. 2020;28(1).

4.      Eklund A, Jensen I, Leboeuf-Yde C, Kongsted A, Jonsson M, Lovgren P, et al. The Nordic Maintenance Care Program: Does psychological profile modify the treatment effect of a preventive manual therapy intervention? A secondary analysis of a pragmatic randomized controlled trial. PLoS One. 2019;14(10):e0223349.

5.      Eklund A, Jensen I, Lohela-Karlsson M, Hagberg J, Leboeuf-Yde C, Kongsted A, et al. The Nordic Maintenance Care program: Effectiveness of chiropractic maintenance care versus symptom-guided treatment for recurrent and persistent low back pain-A pragmatic randomized controlled trial. PLoS One. 2018;13(9):e0203029.

6.      Myhrvold BL, Axén I, Leach MJ, Sundberg T, Gausel AM. Investigating attitudes, skills, and use of evidence-based practice among Norwegian chiropractors; a national cross-sectional study. BMC Health Services Research. 2023.
